# Enhancing Power Conversion Efficiency of Organic Solar Cells with Magnetoplasmonic Fe_3_O_4_@Au@m-ABS Nanoparticles

**DOI:** 10.3390/nano14141175

**Published:** 2024-07-10

**Authors:** Pradeep Kumar, Shih-Han Huang, Chia-Yi Hsu, Ssu-Yung Chung, Hou-Chin Cha, Chih-Min Chuang, Kuen-Lin Chen, Yu-Ching Huang

**Affiliations:** 1 Department of Materials Engineering, Ming Chi University of Technology, New Taipei City 243303, Taiwan; pksingh0021@mail.mcut.edu.tw (P.K.); m11188023@mail2.mcut.edu.tw (S.-Y.C.); hccha@mail.mcut.edu.tw (H.-C.C.); 2 Organic Electronics Research Center, Ming Chi University of Technology, New Taipei City 24301, Taiwan; huangsh@cgu.edu.tw; 3 Center for Sustainability and Energy Technologies, Chang Gung University, Taoyuan 33302, Taiwan; 4 Institute of Nanoscience, National Chung Hsing University, Taichung 40227, Taiwan; jaiganbatte@gmail.com; 5 Department of Physics, National Atomic Research Institute, Taoyuan 325207, Taiwan; cmchuang@nari.org.tw; 6 Department of Physics, National Chung Hsing University, Taichung 40227, Taiwan; 7 Department of Chemical and Materials Engineering, Chang Gung University, Taoyuan 33302, Taiwan

**Keywords:** Fe_3_O_4_@Au@m-ABS, organic photovoltaic, magnetoplasmonic, nanomaterials, solar energy

## Abstract

Organic–inorganic nanocomposites have the potential to be used in photovoltaic materials due to their eco-friendliness, suitable band gaps, and high stability. In this work, we integrated gold and Fe_3_O_4_ magnetic nanoparticles with poly-m-amino benzene sulfonic (m-ABS) to synthesize Fe_3_O_4_@Au@poly-(m-aminobenzenesulfonic acid) (Fe_3_O_4_@Au@m-ABS) magneto-plasmonic nanoparticles (MPNPs) to enhance the performance of the organic photovoltaic (OPV). These MPNPs exhibit broad UV-Vis absorption and a low band gap of 2.878 eV, enhancing their suitability for photovoltaic applications. The MPNPs were introduced into the ZnO electron transporting layer (ETL) and active layer to investigate the influence of MPNPs on the power conversion efficiency (PCE) of the OPVs. When 0.1 vol% MPNPs were incorporated in the ETL, the OPVs achieved a PCE of 14.24% and a fill factor (FF) of 69.10%. On the other hand, when 0.1 vol% MPNPs were incorporated in the active layer, the OPVs showed a PCE of 14.11% and an FF of 68.83%. However, the OPVs without MPNPs only possessed a PCE of 13.15% and an FF of 63.69%. The incorporation of MPNPs increased the PCE by 8.3% in the OPV device. These findings suggest that Fe_3_O_4_@Au@m-ABS MPNPs are promising nanocomposite materials for enhancing the performance of OPVs.

## 1. Introduction

In recent years, due to the rapid development of human civilization, the demand for energy has been increasing daily. However, this has also caused significant environmental damage and pollution. Therefore, seeking clean green energy is one of the most critical challenges for the future. Among the many novel green energy sources, solar cells are a technology with considerable development potential. Solar cells, also known as photovoltaic (PV) cells, are devices that convert light energy directly into electrical energy through the photovoltaic effect. They are a key technology for harnessing solar renewable energy, offering a clean and sustainable alternative to fossil fuels. Solar cells are made from various materials, including silicon, thin films, organic compounds, perovskites, and quantum dots, each aiming to improve efficiency and reduce production costs. When sunlight hits a solar cell, photons are absorbed by the semiconductor material, generating electron–hole pairs that produce electricity. The main components of a solar cell include the semiconductor layer, anti-reflective coating, and electrical contacts, which work together to facilitate the conversion of light into electricity. Solar cells offer numerous advantages, such as being a renewable energy source, being environmentally friendly, and having low operating costs. However, they also face challenges like high initial costs, efficiency limitations, and intermittency issues due to weather and the time of day. Improving the efficiency of solar cells is crucial for overcoming these challenges. High-efficiency solar cells have the potential to become an essential technology for sustainable energy generation. By increasing their efficiency, we can make solar power a more viable and widespread solution, reducing our reliance on fossil fuels and minimizing the environmental impact. The continued development and implementation of advanced materials and technologies in solar cells will play a significant role in achieving this goal, making solar energy a cornerstone of the future energy landscape. 

Several technologies can be used to achieve different levels of efficiency in the design of solar cells. These include thin film technology (23.4% efficiency), multijunction devices (39.2% efficiency), crystalline silicon (c-Si)-based configurations (26.7% theoretical efficiency), perovskite (31% theoretical efficiency), organic thin films (16.4% efficiency), the dye-sensitized method (12.3% efficiency), and perovskite-based quantum dot usage (16.5% efficiency) [1]. Third-generation solar cells are advanced photovoltaic technologies designed to surpass the efficiency and cost limitations of traditional silicon-based and thin-film solar cells. These cells utilize innovative materials and concepts designed to achieve higher efficiencies and lower production costs. Although it is challenging to aim for efficiencies beyond the Shockley–Queisser limit, some next-generation technologies offer unique prospects for significantly improving performance [2]. 

Nanotechnology enables the development of more explicit tools for studying systems, materials, and even the basic components of nature. It creates opportunities for the industrial sector to develop more efficient and innovative manufacturing processes, materials with enhanced qualities, and novel products with applications ranging from electronics to medicine. In recent years, significant progress has been made in polymer-containing iron oxide nanoparticles, resulting in new materials with a wide range of uses. They are good prospects for future technological breakthroughs due to their adjustable features and compatibility with existing manufacturing processes [3]. For example, recent research has indeed demonstrated that the presence of an external magnetic field enhances the transport of charge carriers such as electrons and holes in solar cells. This phenomenon, known as the magneto-photovoltaic effect, has attracted considerable attention in the field of solar cell technology. The behavior of charge carriers is influenced by the presence of magnetic nanoparticles, which may reduce losses and improve the overall solar cell efficiency. This impact is especially noteworthy in organic and hybrid solar cells, where the performance of the device is largely determined by charge carrier mobility and recombination dynamics. It is feasible to improve charge transporting and lower losses by applying magnetic fields optimally, which ultimately results in solar cell systems that are more effective. A potential idea is to use the demagnetization fields produced by MPNPs to regulate the mobility of carriers in solar cells. Comprehending the magnetic characteristics of the materials involved and their interplay with external magnetic fields is vital in understanding the demagnetization impact on the charge carriers, such as (1) the minimum energy spin configuration; (2) exchange field distribution; (3) demagnetization field due to the MPNPs; (4) magnetic moments and magnetization; and (5) carrier mobility and recombination. By understanding these factors and their interplay, researchers can effectively leverage magnetic fields, including demagnetization effects, to control and enhance charge carrier transport in solar cells, ultimately leading to improved device performance and efficiency. Fe_3_O_4_ nanoparticles exhibit ferrimagnetism with an inverse spinel structure [4] and serve as essential building blocks for innovation and advancement across multiple disciplines [5]. Fe_3_O_4_ has a high saturation magnetization and good electrical conductivity due to a phenomenon known as phonon-induced electron tunneling at ambient temperatures (300 K). The assertion that Fe_3_O_4_ nanoparticles can induce an additional Lorentz force on electrons due to their stray magnetic field is correct [4]. 

Recently, Sahar Shabzendedar et al. highlighted the significance of Fe_3_O_4_ composites, such as Fe_3_O_4_/m-ABS, in solar cells [3]. The fabricated device FTO/TiO_2_/m-ABS-Fe_3_O_4_/Al demonstrated an open-circuit voltage (V_oc_) of 0.863 V and a short-circuit current density (J_sc_) of 5.83 mA/cm^2^. However, FF and PCE for this device were reported to be approximately 0.5057 and 4.24%, respectively. Rezaei Bahareh et al. found that the graphene shell around Au nanoparticles (NPs) improved the charge carrier lifetime by 80%, the short-circuit current by 42%, and the efficiency by 100% [6]. This improvement is attributed to the plasmon-induced interfacial charge-transfer transition (PICTT) caused by gold nanoparticles, which enhances the rate of electron generation. Due to their plasmonic properties, Au nanoparticles play a crucial role in enhancing solar cell performance by generating “hot electrons” that reach the outer surface. Here, Fe_3_O_4_ nanoparticles with a spherical structure were synthesized using the hydrothermal method. Additionally, a Fe_3_O_4_@Au core–shell structure was created by coating the Fe_3_O_4_ spherical nanoparticles with a shell layer composed of gold nanoparticles. In this study, we incorporated gold nanoparticles into Fe_3_O_4_ and m-ABS to synthesize a new composite aimed at improving the PCE and FF of OPVs. The resulting composite, Fe_3_O_4_@Au@m-ABS, was introduced into the ZnO and PM6:Y6 active layer of OPVs. The mobility of charge carriers in Fe_3_O_4_@Au@m-ABS was increased due to the electrons emitted by the Au nanoparticles, which significantly contributed to the enhancement of the OPV characteristics. Our results demonstrated that incorporating Fe_3_O_4_@Au@m-ABS nanoparticles into the ZnO ETL increased the PCE from 13.15% to 14.24%. Similarly, the incorporation of these nanoparticles into the active layer enhanced the PCE to 14.11%. This improvement is attributed to the plasmonic effects of gold and the magnetic properties of Fe_3_O_4_, which optimize charge carrier mobility and reduce recombination losses. These findings suggest that magnetic polymer nanocomposites hold significant promise for advancing solar cell technology, offering new pathways to improve efficiency and performance in next-generation solar cells.

## 2. Experiment

### 2.1. Reagents and Materials

Iron (III) chloride hexahydrate (FeCl_3_·6H_2_O), ethylene glycol (EG glycol), sodium acetate (NaAc), polyethylene glycol (PEG), PEI, chloroauric acid (HAuCl_4_), sodium citrate, sodium borohydride (NaBH_4_), m-ABS, and all chemical reagents were commercially available with purity AR and were used directly without further purification. 

### 2.2. Synthesis of Material

Synthesis of Fe_3_O_4_ nanoparticles: The hydrothermal method was employed for the synthesis of Fe_3_O_4_. In detail, a solution of (2.7 g) FeCl_3_·6H_2_O and 70 mL EG was prepared by vigorous stirring. With continuous vigorous stirring, (7.2 g) NaAc and (1.8 g) PEG were added and the prepared solution was transferred to a Teflon-lined stainless-steel autoclave at 200 °C for 8 h. After cooling to room temperature, it was washed three times with deionization water (DIW), and a neodymium iron boron magnet was used (NdFeB magnetic) for magnetic separation. The materials were filtrated to obtain Fe_3_O_4_ spherical nanoparticles less than 400 nm and dry the filtered product to powder. 

Synthesis Fe_3_O_4_@Au nanoparticles: To synthesize Fe_3_O_4_@Au, first we prepared a solution of Fe_3_O_4_ and PEI. So, 50 mL of 5 mg/mL PEI solution was sonicated for 2 h. Then, 0.2 gm of dried Fe_3_O_4_ powder was added to the PEI solution and subjected to ultrasonic vibration for two hours. In the second step, we synthesized the Au seeds. A solution of 2 mL HAUCl_4_ (1% wt) and 180 mL DI water was stirred for 1 min. Then, 4 mL of 38.8 mM sodium citrate was added and stirred for 1 min more. After this, 2 mL NaBH_4_ (0.075% wt) was mixed and stirred for 5 min. During this reaction, the color changed immediately from yellow to red, obtaining 10 nm Au seeds. This solution was placed in a dark room. 

In the final step, both of the prepared solutions were mixed and shaken for 2 h. A solution of 0.5 gm PEI (after 2 h of sonication) was added and shaken for 20 min. Then, this solution was heated in an oven at 60 °C for 2 h and cooled down in a dark room overnight. It was centrifuged at 6000 rpm for 30 min and synthesized Fe_3_O_4_@Au nanoparticles were obtained. 

**Figure 1:**
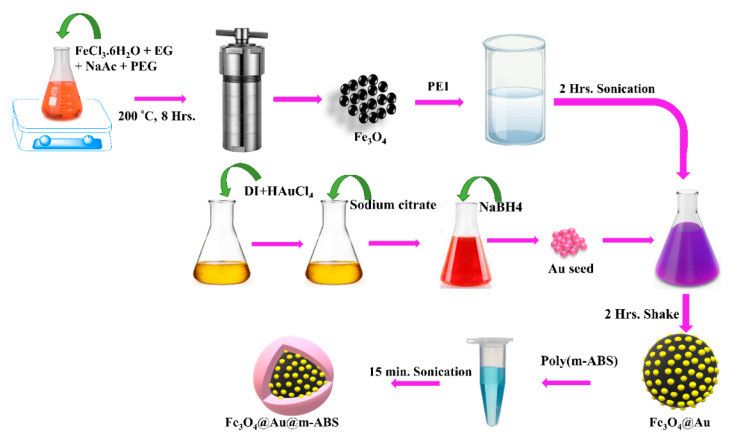
Schematic illustration of the process for the synthesis of Fe_3_O_4_@Au@m-ABS nanoparticles.

Synthesis of Fe_3_O_4_@Au@m-ABS magneto-plasmonic nanoparticles: To synthesize Fe_3_O_4_@Au@m-ABS MPNPs, first we prepared a solution of 0.002 gm m-ABS and ultrasonically vibrated this for 15 min. m-ABS solution and solution of Fe_3_O_4_@Au were mixed and sonicated for 15 min. Then, it was left to react for 30 min. It was centrifuged at 12,000 rpm for 30 min to obtain the Fe_3_O_4_Au-@m-ABS nanoparticles. The schematic diagram is shown in Figure 1.

### 2.3. Fabrication of Organic Solar Cells

Materials: Zinc acetate powder was purchased from Thermo Scientific, while poly[[4,8-bis [5-(2-ethylhexyl)-4-fluoro-2-thienyl]benzo [1,2-b:4,5-b’]dithiophene-2,6-diyl]-2,5-thiophenediyl [5,7-bis(2-ethylhexyl)-4,8-dioxo-4H,8H-benzo [1,2-c:4,5-c’]dithiophene-1,3-diyl]-2,5-thiophenediyl] (PM6) and 2,2′-[[12,13-Bis(2-ethylhexyl)-12,13-dihydro-3,9-diundecylbisthieno [2′‘,3′‘:4′,5′]thieno [2′,3′:4,5]pyrrolo [3,2-e:2′,3′-g][2,1,3]benzothiadiazole-2,10-diyl]bis[methylidyne(5,6-difluoro-3-oxo-1H-indene-2,1(3H)-diylidene)]]bis[propanedinitrile] (Y6) were obtained from 1-Materials. Molybdenum oxide (MoO_X_, 99.9995%) was purchased from Alfa Aesar, and Ag was purchased from Gredmamann Taiwan Ltd., Taipei, Taiwan. All chemicals were used as received without any further treatment. The molecular structure of PM6 and Y6 is shown in Figure S1.

Device Fabrication: The reference cells of OPVs were fabricated with an inverted structure as follows: glass/ITO/ZnO/PM6:Y6/MoO_X_/Ag. The substrates used were LT ITO (15 ohm). Prior to the deposition of the ZnO film, pre-patterned and ITO-covered glass substrates were cleaned sequentially with glass cleaner, acetone, and isopropanol in an ultrasonic bath for 30, 10, and 10 min, respectively, followed by a 10 min surface treatment with oxygen plasma. For the sol–gel ZnO solution, 50 mg of zinc acetate powder was dissolved in 1000 μL of 2-methoxyethanol (2-ME) and 14 μL of ethanolamine (EA). The ZnO solution was filtered through a 0.45 μm filter and spin-coated onto the substrate at 3000 rpm for 30 s, followed by annealing on a hotplate at 180 °C for 20 min under ambient conditions. The following were completed in a nitrogen-filled glovebox. The PM6:Y6 solution was prepared at a ratio of 1:1.2, with a total concentration of 17.6 mg/mL in chloroform (CF). One volume percent (1 vol%) of 1-chloronaphthalene (CN) was added as an additive, and the mixture was stirred at 40 °C for 1 h prior to use. The spin-coated sol–gel ZnO samples were transferred to a nitrogen-filled glove box, and the PM6:Y6 solution was dropped onto the spin-coated ZnO sample at a speed of 5000 rpm; this was continued for 30 s to complete the coating process. Finally, the hole transport layer of the MoO_X_ and Ag electrode was deposited using thermal vapor evaporation under a vacuum degree of 2 × 10^−6^ Torr, with deposition rates of 0.1 Å/s and 1.5 Å/s and thicknesses of 5 nm and 100 nm, respectively. The device active area was 0.04 cm^2^. To investigate the effect of MPNPs in the ETL and active layer, MPNPs were added to the ZnO solution and the PM6:Y6 solution at ratios of 0.1 vol% and 0.2 vol%, respectively.

Material and Device Characterization: Measurements were performed using a voltage source meter (Keithley 2410) under an AM 1.5G solar simulator with irradiation at 100 mW/cm^2^. External quantum efficiency (EQE) was performed by a system (QE-R_PV, Enlitech, Kaohsiung, Taiwan). Time-resolved PL (TRPL) was performed by exciting samples with a 532 nm diode laser (LDH-P-C-405, PicoQuant, Berlin, Germany). The TRPL was recorded by a system (UniDRON-plus, UniNano Tech, Yongin, Republic of Korea). Transient photovoltage decay (TPV) and transient photocurrent decay (TPC) analysis measurements of PSCs were recorded with Paios (Fluxim, electrical measurement all-in-one platform for solar cells). The surface morphology of the film was studied by SEM (JSM-7800F, Jeol, Tokyo, Japan) and atomic force microscopy (AFM) (Dimension-3100 Multimode, Digital Instruments, Plainview, NY, USA). UV-Vis absorption spectra were measured by JASCO U-550. The work function values of films on ITO electrodes were obtained using a KP 6500 Digital Kelvin probe (McAllister Technical Services, Co., Coeur d’Alene, ID, USA).

## 3. Results and Discussion

### 3.1. The Morphology and Optical Property Analysis

**Figure 2:**
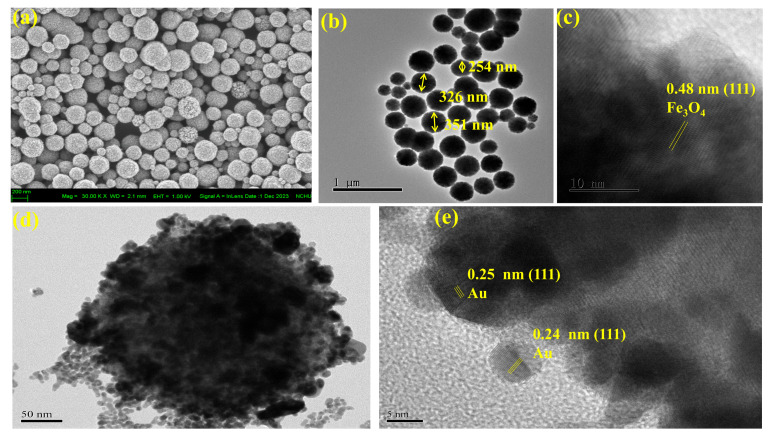
The morphological characteristics of Fe_3_O_4_ (**a**) were examined using FE-SEM. High-magnification cross-section TEM images of (**b**,**c**) Fe_3_O_4_ and (**d**,**e**) Fe_3_O_4_@Au.

The surface morphology of Fe_3_O_4_ nanoparticles was confirmed by the FE-SEM (JEOL JSM-7800F). The synthesized Fe_3_O_4_ nanoparticles with similar shapes and sizes were examined from the profile image, as delineated in Figure 2a. The view of the profile looked like spherical-shaped Fe_3_O_4_ nanoparticles (Figure 2a). Moreover, Figure 2b examined the spherical shapes of the Fe_3_O_4_ by using a transmission electron microscope (TEM). Fe_3_O_4_ has (111) a crystal face with a d-spacing of 0.48 nm, as illustrated in Figure 2c [7]. However, Fe_3_O_4_@Au nanoparticles have a core–shell structure, as mentioned in Figure 2d. In the examination of TEM results for Fe_3_O_4_@Au, it was found that the gold nanoparticles fully cover the particle surface of Fe_3_O_4_ nanoparticles, as seen in Figure 2d–e. Au nanoparticles have a d-spacing of 0.24 nm and 0.25 nm with a lattice plane (111), as illustrated in Figure 2e [8,9]. This is believed to be because the gold covers the iron oxide. The particle sizes and d-spacing of Fe_3_O_4_ and Fe_3_O_4_@Au were calculated by using ImageJ software (1.54h) [10].

**Figure 3:**
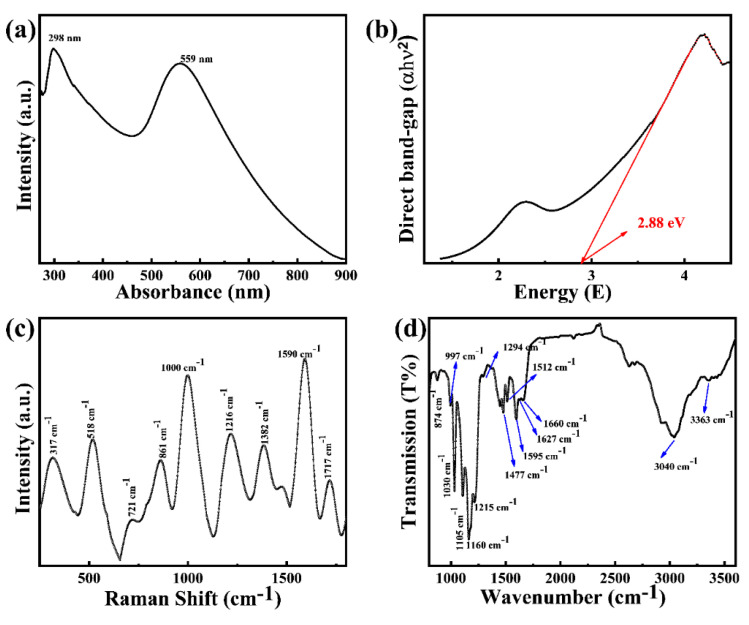
Optical properties of Fe_3_O_4_@Au@m-ABS. (**a**) UV-Vis absorbance. (**b**) Direct and indirect band gap. (**c**) Raman vibration mode. (**d**) FT-IR vibrations.

The absorbance peaks of Fe_3_O_4_@Au@m-ABS nanoparticles were analyzed with UV-Vis (JASCO U-550) spectroscopy, as shown in Figure 3a. The liquid form of Fe_3_O_4_@Au@m-ABS nanoparticles was poured onto glass and dried at room temperature. After drying, a film of nanoparticles was formed on the glass, which was used to check the UV absorbance. The absorbance peak at 298 occurred due to the π-π* transition of the benzenoid moiety of m-ABS. The intense absorption peak at 599 nm originates from the localized surface plasmon resonance of gold [11,12]. Moreover, the broader peak at 559 nm occurred due to the overlap of Au absorbance and polaronic transition of m-ABS absorbance [13]. Madhurima Das el. evaluated the direct band gap m-ABS with a value of 3.13 eV [13], while, in this experiment, the lower direct band gaps of Fe_3_O_4_@Au@m-ABS nanoparticles were calculated and found to be 2.88 eV, as illustrated in Figure 3b. As a result of the reduction in the band gap, the compound has dielectric confinement [14], which allows it to absorb photons fast and without being disturbed [15,16]. Thus, it might result in future applications in optoelectronics and nanoelectronics [17,18,19]. The atomic vibrations were studied using Raman spectroscopy (NANOSCOPE/NS220), as shown in Figure 3c. In Fe_3_O_4_, we identified Raman-active modes at 317 cm^−1^, 518 cm^−1^, 721 cm^−1^, and 1382 cm^−1^ [9]. The peak around 1590 cm^−1^ belongs to Au [9,11,20,21,22], while the Raman vibrations that peaked around 861 cm^−1^, 1000 cm^−1^, 1216cm^−1^, 1717 cm^−1^, and 721 cm^−1^ 4-ABS were assigned to m-ABS. Moreover, Fe_3_O_4_@Au@m-ABS nanoparticles were confirmed by the FT-IR (JASCO-FT-IR) spectrum in Figure 3d. The broad peak at 3363 cm^−1^ was assigned to N–H stretching vibration in the secondary amine and the peak at 1627 cm^−1^ was attributed to O-H bending vibration in water molecules or allocated to Raman-active –C=C– ring stretching vibration [23]. The peaks at 1512 and 1477 cm^−1^ were assigned to the benzenoid vibration and the peak at 1595 cm^−1^ corresponded to the C=C stretching vibration of quinoid [24,25]. The presence of broadening at a wavenumber higher than 200 cm^−1^ was typical of the conducting from complect doping. This demonstrates that the quinone and benzenoid rings have been combined and, in fact, they have almost been united because of the resonance phenomenon and doping process. The peak at 1294 cm^−1^ was assigned to C–N stretching vibration and it established a pie–electron delocalization in the system. Also, the peaks at 1215 cm^−1^ and 1160 cm^−1^ were attributed to vibrations of the C–N^•+^ group and –NH^•+^ group, respectively [23]. The former band is a specific band in the conducting protonated and doping polymer. The presence of SO_3_H groups can be confirmed with both bands at 1160 cm^−1^ for O=S=O stretching vibrations [24]. The band at 1105 cm^−1^ was the characteristic peak of the C–N stretching mode [24]. The peaks at 874 cm^−1^, 997 cm^−1^, and 1030 cm^−1^ were assigned to Fe–O vibrations. We assume that the reduction of Au^3+^ to Au^0^ results in strong bands at 3040 cm^−1^ (C–H stretch in an aromatic ring) [25,26].

### 3.2. Organic Photovoltaic Characteristics

**Figure 4:**
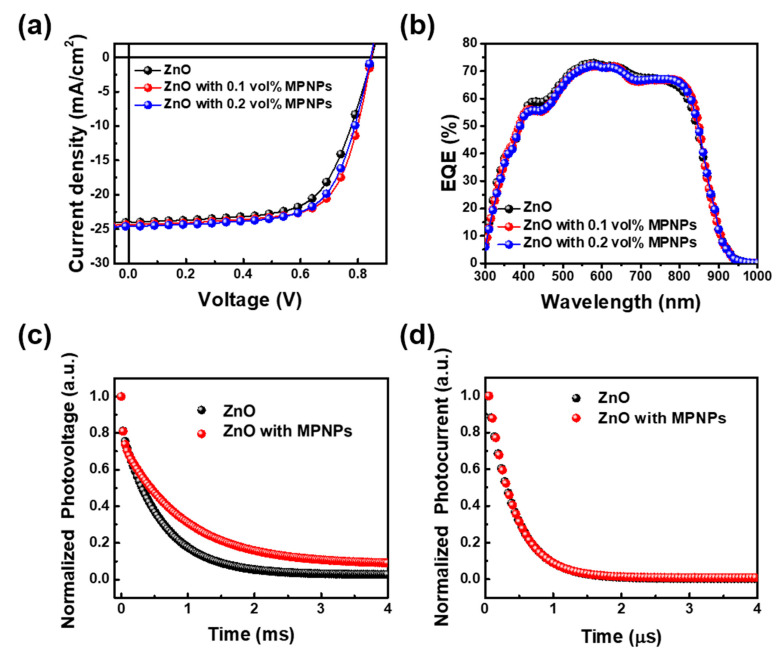
Device characterization prepared using ZnO films with and without MPNPs. (**a**) J-V curves. (**b**) EQE measurement. (**c**) TPV. (**d**) TPC.

In the study of OPVs, an inverted device structure of ITO/ZnO/PM6:Y6/MoO_X_/Ag was utilized, where ZnO served as the ETL and the mixture of PM6 and Y6 functioned as the active layer. Here, MoO_X_ and ZnO serve as crucial components due to their properties as n-type semiconductors with band gaps around 3.2 eV [27] and 3.37 eV [28], respectively. These materials are known for their high adsorption efficiency and excellent thermal and mechanical stability. The work function of MoO_X_, which normally ranges from 5.3 to 5.63 eV in the ZnO/PM6/MoO_X_ structure [27], is similar to that of the bottom-layer ZnO at 5.2 eV. Electron transport from the photoactive layer (PM6) to MoO_X_ depends on band bending at the interface between MoO_X_ layers and the active layer, which is caused by this closeness in work function. To further evaluate the plasmonic effect in organic solar cells, the MPNPs were introduced into the ETL of ZnO and the active layer, respectively. The plasmonic effect on the ETL was investigated first. Fe_3_O_4_@Au@m-ABS nanoparticles with different concentrations of 0.1 vol% and 0.2 vol% MPNPs were added into ZnO solution to prepare the ZnO ETL film. The current density–voltage (J-V) curves, statistical distribution, and photovoltaic parameters of OPVs using ZnO with and without MPNPs are shown in Figure 4a and Figure S2 and listed in Table 1, respectively. The OPV prepared using pristine ZnO shows an open-circuit voltage (V_oc_) of 0.85 V, a short-circuit current density (J_sc_) of 23.98 mA/cm^2^, and an FF of 0.646, resulting in an average PCE of 12.65%, of which the highest PCE was 13.15%. For the ZnO film with 0.1 vol% MPNPs, OPVs demonstrated an average PCE of 14.24%, accompanied by a V_oc_ of 0.85 V, a J_sc_ of 24.35 mA/cm^2^, and a FF of 0.691. This is attributed to the addition of 0.1 vol% MPNPs in ZnO. The J_sc_ enhancement of OPVs corresponded to the EQE measurement in Figure 4b. However, increasing the concentration of MPNPs to 0.2 vol% slightly reduced the PCE of the OPVs due to a decrease in FF compared to the 0.1 vol% MPNPs condition. To understand the effect of MPNPs in ETL, we characterized ZnO with 0.1 vol% MPNPs. In Figure 4c,d, TPV and TPC were used to obtain the carrier lifetime and extraction lifetime in the OPVs. In TPV measurements, the carrier lifetime of the OPVs, prepared using pristine ZnO, was 591 μs. For the ZnO with MPNPs, the carrier lifetime of OPVs was 715 μs. The extended lifetime observed with the addition of MPNPs in ZnO indicated reduced carrier recombination in the OPVs. In the TPC measurement, the carrier extraction lifetimes of OPV using ZnO with and without MPNPs were 0.512 μs and 0.507 μs, respectively. The OPVs prepared using the ZnO film with MPNPs show the lowest carrier extraction lifetimes, attributed to the Fe_3_O_4_@Au@m-ABS doping in the ZnO film, which correlates with the improved device performance.

**Figure 5:**
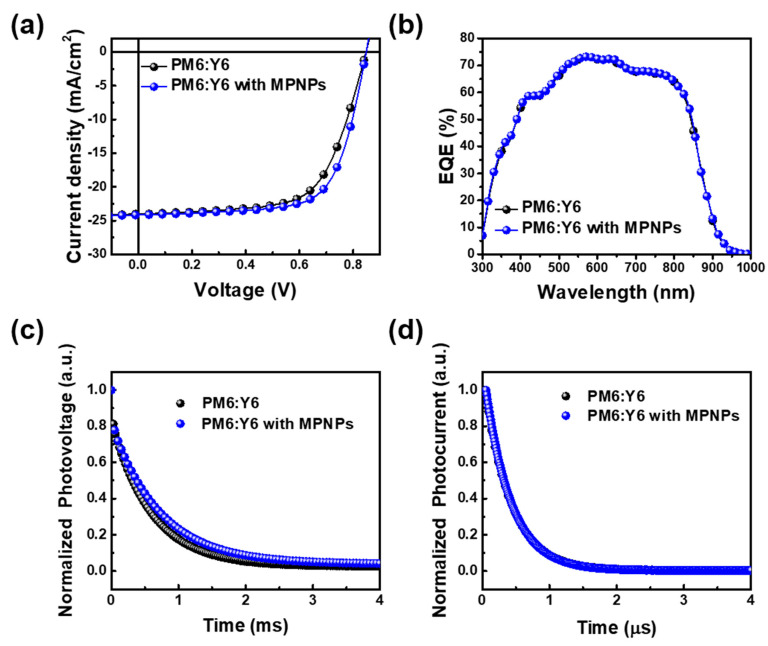
Device characterization was prepared using PM6:Y6 films with and without MPNPs. (**a**) J-V curves. (**b**) EQE measurement. (**c**) TPV. (**d**) TPC.

A concentration of 0.1 vol% MPNPs in the ETL showed a promising enhancement in the properties of OPV devices. To further understand the impact of MPNPs on OPV performance, we also investigated their effect when incorporated into the active layer. Specifically, MPNPs with a concentration of 0.1 vol% were integrated into the active layer solution. The statistical distribution, device performance, and J-V curves of OPVs with and without MPNPs in the active layer are shown in Figure S3 and Figure 5a and Table 1. The OPVs prepared using the pristine active layer exhibit a V_oc_ of 0.85 V, a J_sc_ of 23.98 mA/cm^2^, and an FF of 0.646, resulting in an average PCE of 12.65%, of which the highest PCE was 13.15%. For the active layer with 0.1 vol% MPNPs, the OPVs showed an average PCE of 14.11%, accompanied by a Voc of 0.85V, a J_sc_ of 24.16 mA/cm^2^, and an FF of 0.688. This notable enhancement is primarily attributed to the significant improvement in the FF, which is due to the addition of 0.1 vol% MPNPs in the active layer. In Figure 5b, the EQE measurement does not show obvious improvement for the addition of MPNP in the active layer, indicating that the primary enhancement mechanism is related to factors other than EQE. TPV measurements, as illustrated in Figure 5c, were used to evaluate the carrier lifetime of OPVs prepared with MPNPs in the active layer. The carrier lifetime significantly increased from 591 μs to 902 μs with the addition of MPNPs, indicating reduced carrier recombination. For the TPC measurement, the carrier extraction time of OPVs slightly decreased from 0.512 μs to 0.508 μs with the addition of MPNPs in the active layer, as shown in Figure 5d. This suggests that the overall improvement in OPV performance can be attributed to enhanced charge carrier dynamics, including both increased carrier lifetime and slightly faster carrier extraction times, due to the incorporation of MPNPs into the active layer.

We further characterized the morphology, hydrophilicity, and energy level of films without and with MPNPs. AFM images of the ZnO and active layers for morphological characterization are shown in Figure S4 and Table S1. The roughness of ZnO films and PM6:Y6 films slightly increased with the addition of MPNPs. In the hydrophilicity measurement (Figure S5), the modified films and pristine films did not show significant differences for both ZnO and PM6:Y6 films. The energy levels of the films were determined using a Kelvin probe measurement to obtain their work function, which corresponded with the results from the UPS measurement [29]. The contact potential difference (CPD), representing the work function difference between the reference tip and the sample, is shown in Table S1. With the addition of MPNPs, the CPD values of ZnO film did not exhibit significant shifts. However, for the PM6:Y6 active layer, the CPD values shifted upon the addition of MPNPs, indicating that the effect of MPNPs on PM6:Y6 films was due to changes in the work function.

The enhancement in device performance is attributed to the addition of MPNPs, which have magnetoplasmonic properties. In the electron transporting layer, the magnetic field of MPNPs facilitates the quiescent discharge of electrons, resulting in an increased photocurrent in OPVs [30]. This result is consistent with the TPV measurement. In the active layer, the magneto MPNPs induce the dipoles of the active layer, which promotes the effective exciton dissociation [31,32]. The shell structure of MPNPs contains sulfonyl functional groups, which demonstrate the tunability of the work function at the interface between the active layer and the subsequent HTL [33]. This work function shift improves hole carrier transport.

## 4. Conclusions

In this study, Fe_3_O_4_ spherical nanoparticles were used to synthesize hybrid magnetic nanoparticles (Fe_3_O_4_@Au@m-ABS) to enhance OPV performance. The UV-Vis absorption peak at 298 nm was related to m-ABS, while the broad UV-Vis absorption peak at 559 nm was due to the plasmonic behavior of gold nanoparticles, with a band gap of 2.88 eV using Tauc Plot. The incorporation of these magnetic and plasmonic nanocomposites into the ZnO of ETL and the active layer, as PM6:Y6, of OPV devices significantly improved their performance. Specifically, adding 0.1 vol% of Fe_3_O_4_@Au@m-ABS nanoparticles to the ZnO ETL increased the average PCE from 12.65% to 14.24%. Similarly, incorporating these nanoparticles into the active layer enhanced the PCE to 14.11%. These improvements are attributed to the better charge carrier mobility and reduced recombination losses driven by the plasmonic effects of gold and the magnetic properties of Fe_3_O_4_. The enhanced mobility and reduced recombination losses suggest that such nanocomposites can play a crucial role in the development of high-efficiency solar cells. Furthermore, the ability to finely tune the optical and electronic properties of these materials through the strategic incorporation of magnetic and plasmonic components presents a promising avenue for further research and development in the field of photovoltaics. These findings underscore the potential of magnetic polymer nanocomposites in advancing solar cell technology by offering new pathways to enhance performance in next-generation photovoltaic devices.

## Figures and Tables

**Table 1:** Device performance of OPVs prepared using ZnO and PM6:Y6 films at different concentrations of MPNP.

Sample	Concentration of MPNP (vol%)	V_OC_ (V)	J_SC_ (mA/cm^2^)	FF (%)	PCE (%)
Reference	0	0.85 (0.84 ± 0.00)	23.98 (23.66 ± 0.25)	64.69 (63.63 ± 1.96)	13.15 (12.65 ± 0.38)
ZnO	0.1	0.85 (0.84 ± 0.00)	24.35 (24.29 ± 0.19)	69.10 (68.30 ± 0.67)	14.24 (13.91 ± 0.20)
	0.2	0.84 (0.83 ± 0.01)	24.61 (24.17 ± 0.20)	67.17 (65.71 ± 1.29)	13.96 (13.26 ± 0.44)
PM6:Y6	0.1	0.85 (0.84 ± 0.01)	24.16 (24.29 ± 0.12)	68.83 (66.33 ± 2.52)	14.11 (13.49 ± 0.57)

## Data Availability

Data is contained within the article and Supplementary Material.

## Supplementary Materials

The following supporting information can be downloaded at: https://www.mdpi.com/article/10.3390/nano14141175/s1, Figure S1. The molecular structure of Y6 and PM6. Figure S2. Statistical distribution of OPVs prepared using ZnO films with various amounts of MPNPs. Figure S3. Statistical distribution of OPVs prepared using PM6:Y6 films with various amounts of MPNPs. Figure S4. Morphology characterization of films (a) pristine ZnO film, (b) ZnO:MPNPs film, (c) pristine PM6:Y6 film and (d) PM6:Y6:MPNPs film. Table S1. The roughness and CPD of the ZnO and PM6:Y6 films without and with MPNPs. Figure S5. Hydrophilicity characterization of the films: (a) pristine ZnO film, (b) ZnO:MPNPs film, (c) pristine PM6:Y6 film, and (d) PM6:Y6:MPNPs film.
