# Supplementary material for: Enhancing Power Conversion Efficiency of Organic Solar Cells with Magnetoplasmonic Fe3O4@Au@m-ABS Nanoparticles
Source: Nanomaterials (Basel). 2024 Jul 10;14(14):1175. doi: 10.3390/nano14141175 (PMC11279951; doi:10.3390/nano14141175)
Supplement: Supplementary file 1 [file nanomaterials-14-01175-s001.zip › nanomaterials-3073041-supplementary.pdf]

Enhancing Power Conversion Efficiency of Organic Solar Cells with Magnetoplasmonic Fe_3O_4@Au@m-ABS Nanoparticles

Pradeep Kumar[1†], Shih-Han Huang[2,3†], Chia-Yi Hsu[4], Ssu-yung Chung[1], Hou-Chin Cha[1,2], Chih-Min Chuang[5], Kuen-Lin Chen[4,6*], Yu-Ching Huang[1,2,3,7*]

[1]Department of Materials Engineering, Ming Chi University of Technology, New Taipei City 243303, Taiwan

[2]Organic Electronics Research Center, Ming Chi University of Technology, New Taipei City 24301, Taiwan

[3]Center for Sustainability and Energy Technologies, Chang Gung University, Taoyuan 33302, Taiwan

[4]Institute of Nanoscience, National Chung Hsing University, Taichung 40227, Taiwan

[5]Department of Physics, National Atomic Research Institute, Taoyuan 325207, Taiwan

[6]Department of Physics, National Chung Hsing University, Taichung 40227, Taiwan

[7]Department of Chemical and Materials Engineering, Chang Gung University, Taoyuan 33302, Taiwan

[†]These authors contributed equally to this work

**Corresponding authors: klchen@phys.nchu.edu.tw (K.-L. Chen) and huangyc@mail.mcut.edu.tw (Y.-C. Huang)*

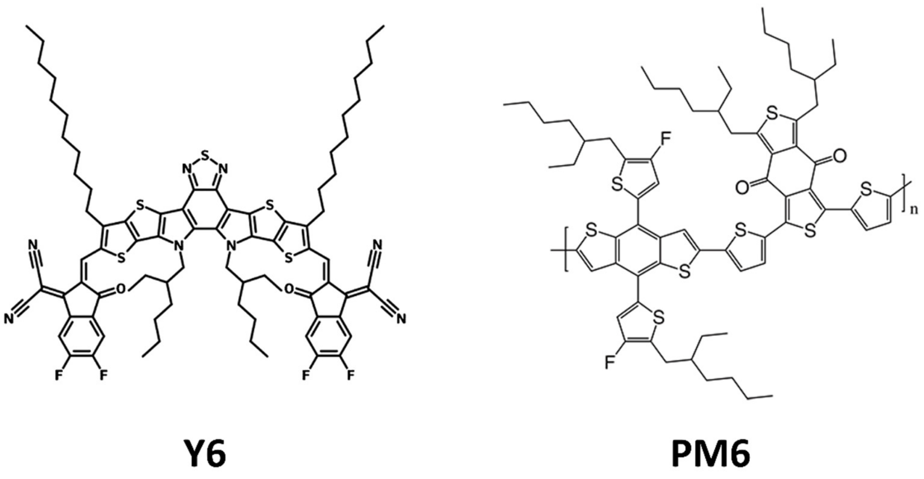

Figure S1. The molecular structure of Y6 and PM6.

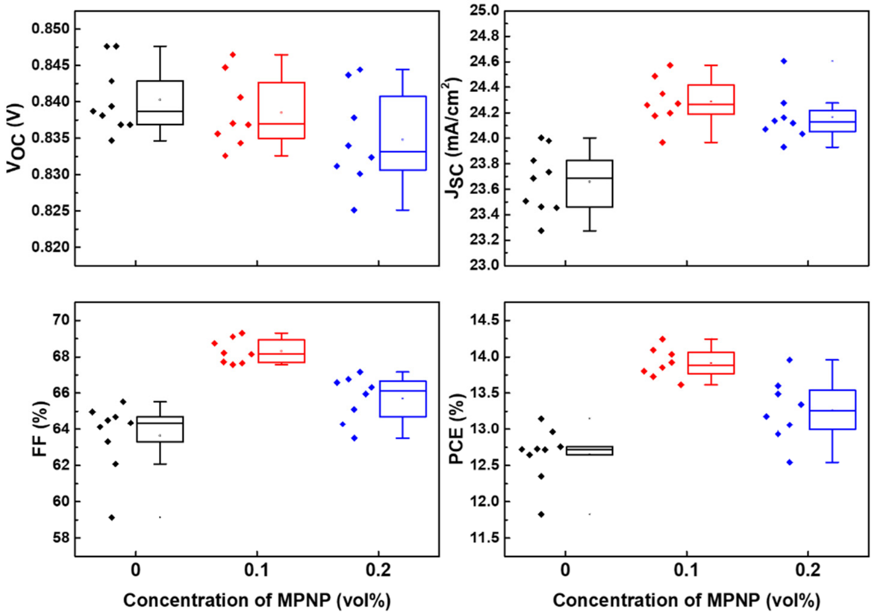

Figure S2. Statistical distribution of OPVs prepared using ZnO films with various amounts of MPNPs.

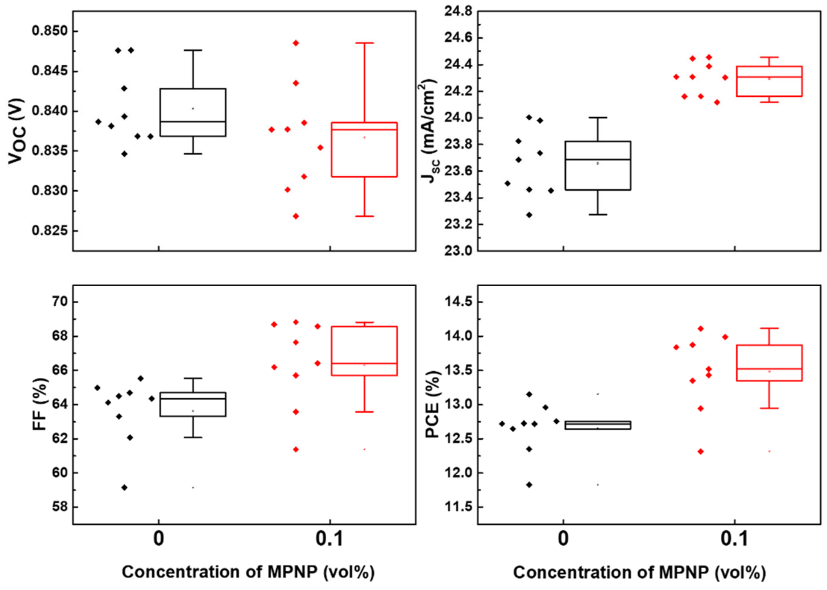

Figure S3. Statistical distribution of OPVs prepared using PM6:Y6 films with various amounts of MPNPs.

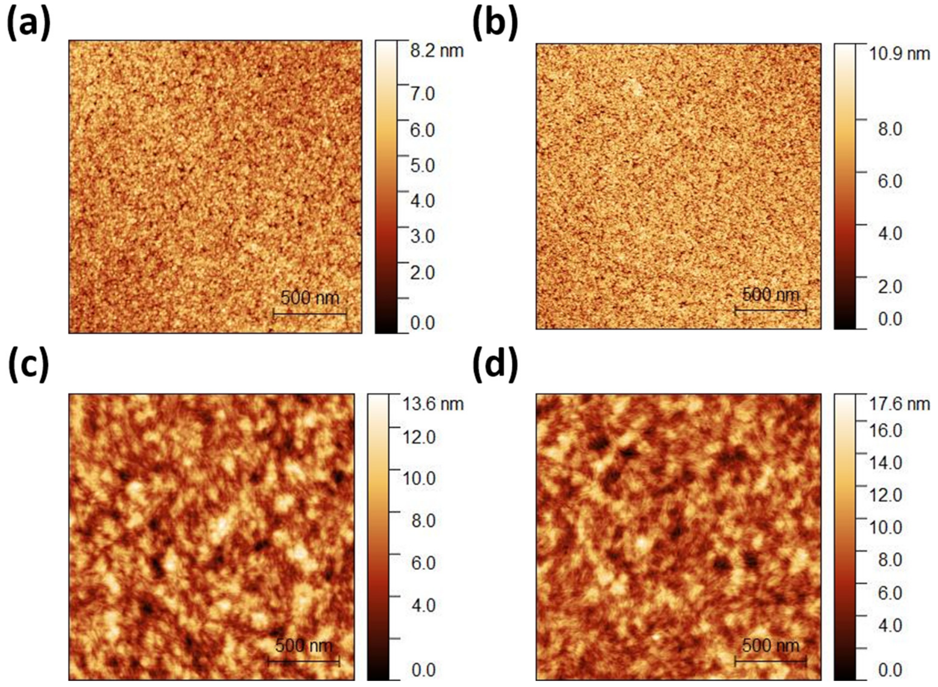

Figure S4. Morphology characterization of f films (a) pristine ZnO film, (b) ZnO:MPNPs film, (c) pristine PM6:Y6 film and (d) PM6:Y6:MPNPs film.

Table S1. The roughness and CPD of the ZnO and PM6:Y6 films without and with MPNPs

Film	Roughness (nm)	CPD (mV)
ZnO	0.89	0.235
ZnO:MPNPs	1.28	0.234
PM6:Y6	1.96	0.311
PM6:Y6:MPNPs	2.32	0.340

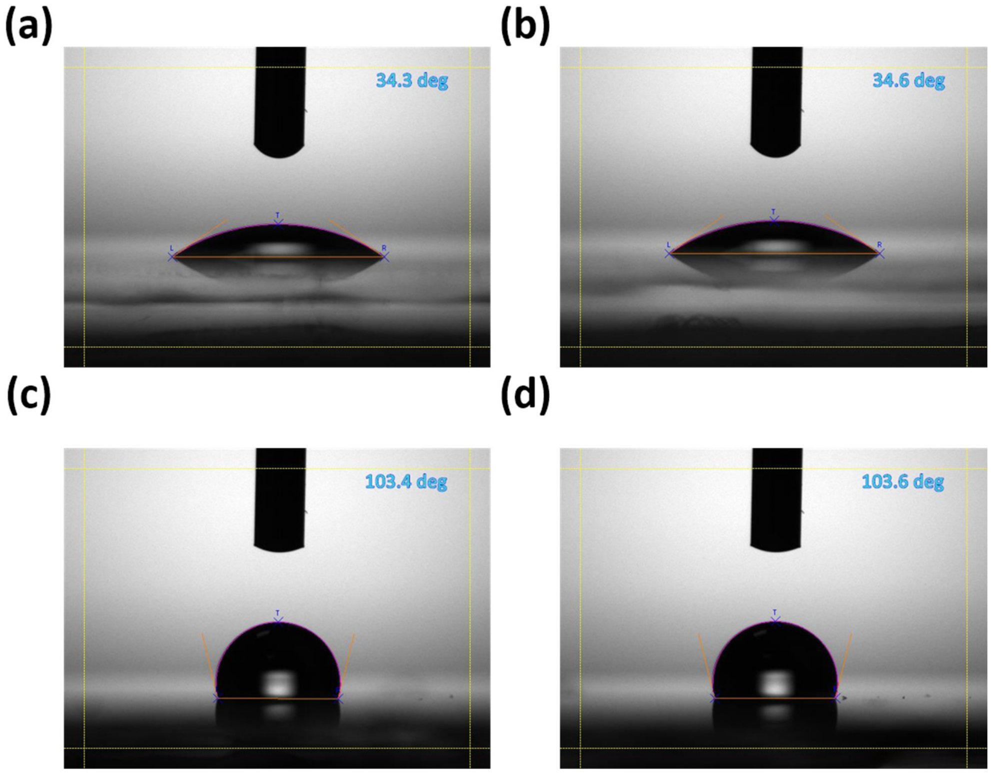

Figure S5. Hydrophilicity characterization of the films: (a) pristine ZnO film, (b) ZnO:MPNPs film, (c) pristine PM6:Y6 film, and (d) PM6:Y6:MPNPs film.
